# Primary Sjögren’s Syndrome Presenting With Severe Hypokalemia Due to Distal Renal Tubular Acidosis: A Case Report

**DOI:** 10.7759/cureus.100325

**Published:** 2025-12-29

**Authors:** Ngoc Quy Nguyen, Dao A Dang, Tien T Le, The T Le, Son V Nguyen

**Affiliations:** 1 Department of Nephrology and Endocrinology, Da Nang Hospital, Da Nang, VNM; 2 Department of Stroke, Da Nang Hospital, Da Nang, VNM

**Keywords:** autoimmune disease, distal renal tubular acidosis, electrolyte imbalance, hypokalemia, renal involvement, sjögren’s syndrome

## Abstract

Primary Sjögren’s syndrome is a systemic autoimmune disorder with diverse clinical manifestations. Although exocrine gland dysfunction is the hallmark, renal involvement, particularly distal renal tubular acidosis (type 1 RTA), can occur and may precede classic sicca symptoms. We report a case of a 55-year-old woman presenting with acute lower limb weakness due to severe hypokalemia. Laboratory investigations revealed normal anion gap metabolic acidosis with inappropriately alkaline urine, consistent with type 1 RTA. Further evaluation uncovered subtle sicca symptoms, parotid gland enlargement, and serologic positivity for anti-Ro/SSA and anti-La/SSB antibodies, supporting the diagnosis of primary Sjögren’s syndrome. The patient responded well to potassium and bicarbonate supplementation. This case underscores the importance of considering autoimmune etiologies such as primary Sjögren’s syndrome in patients with unexplained hypokalemia and tubular acidosis, even in the absence of overt sicca features.

## Introduction

Sjögren’s syndrome is an autoimmune disorder characterized by lymphocytic infiltration of exocrine glands, resulting in sicca symptoms and a wide range of systemic manifestations. It can occur as a primary disorder or secondary to other autoimmune diseases [1]. Most patients exhibit impaired exocrine gland function, leading to symptoms such as dry eyes and dry mouth, which may cause difficulty swallowing and an increased risk of dental caries. Physical examination may reveal non-tender parotid gland enlargement. Approximately 30% to 40% of patients present with systemic manifestations, and renal involvement occurs in about 5% of cases [2]. The pathophysiological mechanisms linking Sjögren’s syndrome to distal renal tubular acidosis (RTA) remain incompletely understood. Proposed mechanisms include loss of H⁺-ATPase expression in renal tubular cells and the presence of autoantibodies against carbonic anhydrase II in patients with primary Sjögren’s syndrome. In this report, we present a case of suspected primary Sjögren’s syndrome initially manifesting as hypokalemia due to a renal tubular defect. This case highlights the clinical heterogeneity of Sjögren’s syndrome and the importance of a systematic approach to hypokalemia, a common electrolyte disturbance in clinical practice.

## Case presentation

A 55-year-old female patient with a history of bile duct stones treated via endoscopic retrograde cholangiopancreatography, no prior head and neck irradiation, and a prior episode of mild hypokalemia managed with oral potassium supplementation presented with acute lower limb weakness. One day prior to admission, she noticed progressive weakness in both legs. She self-medicated with an unknown drug, and by the morning of admission, the weakness had acutely worsened and spread to the upper limbs and lower back, prompting her admission to the ICU. At the ICU, she was found to have severe hypokalemia (1.66 mmol/L) and received intravenous potassium supplementation. Once stabilized, she was transferred to the nephrology ward for further evaluation.

On examination at the nephrology unit, the patient was alert and oriented, with no edema or paresthesia. Muscle strength was 5/5 throughout. She denied nausea, headache, or focal neurologic signs. Her pulse was 78 beats per minute (bpm), and her blood pressure was 130/80 mmHg. She was 155 cm tall and weighed 58 kg, with a corresponding BMI of 24.2 kg/m². The patient reported a normal diet, with no dietary restrictions. Further history-taking revealed symptoms suggestive of sicca syndrome, including eye dryness when exposed to wind and difficulty swallowing dry foods. On examination, the right parotid gland was non-tender and enlarged.

**Figure 1:**
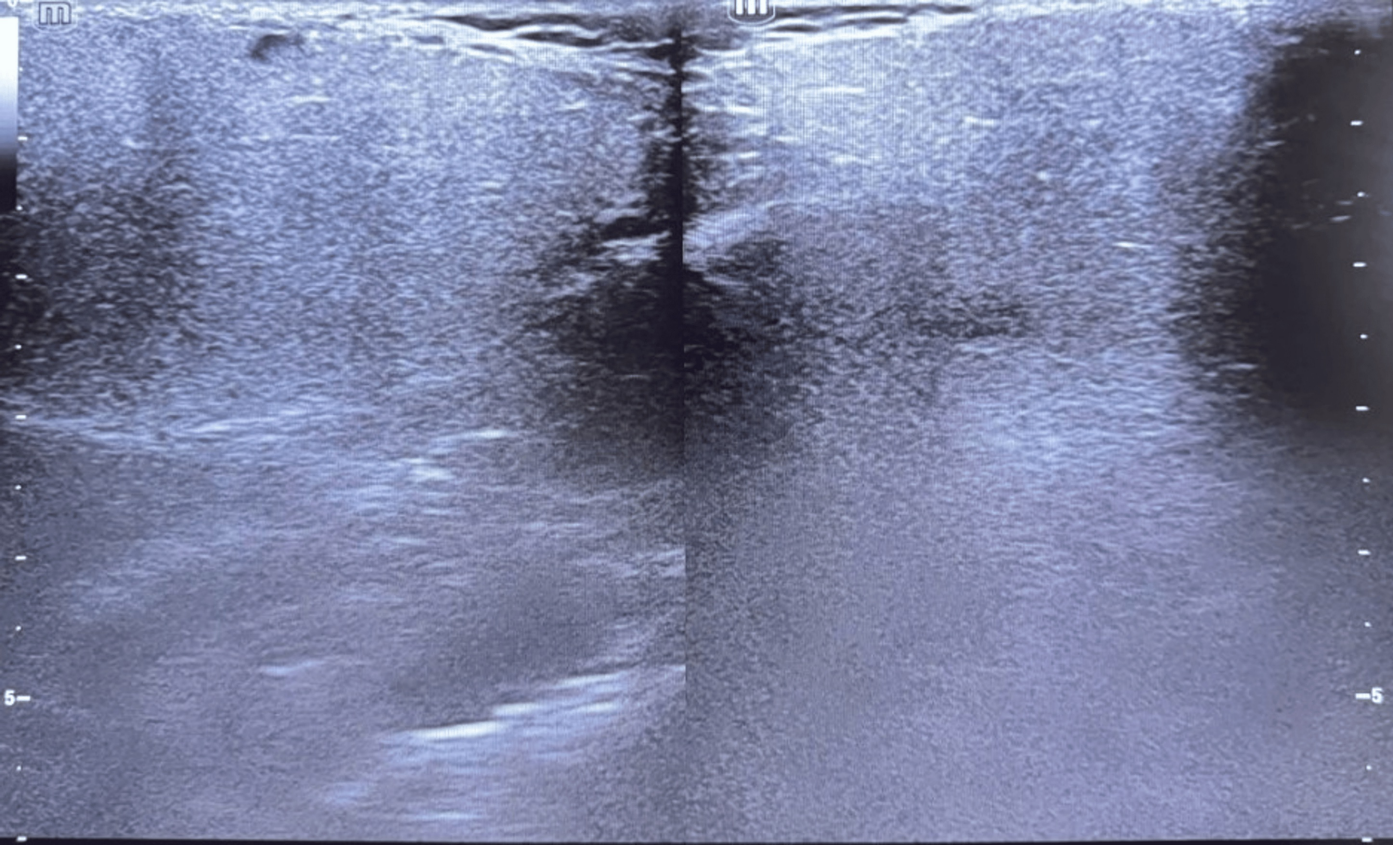
Ultrasonography of the right parotid gland

**Figure 2:**
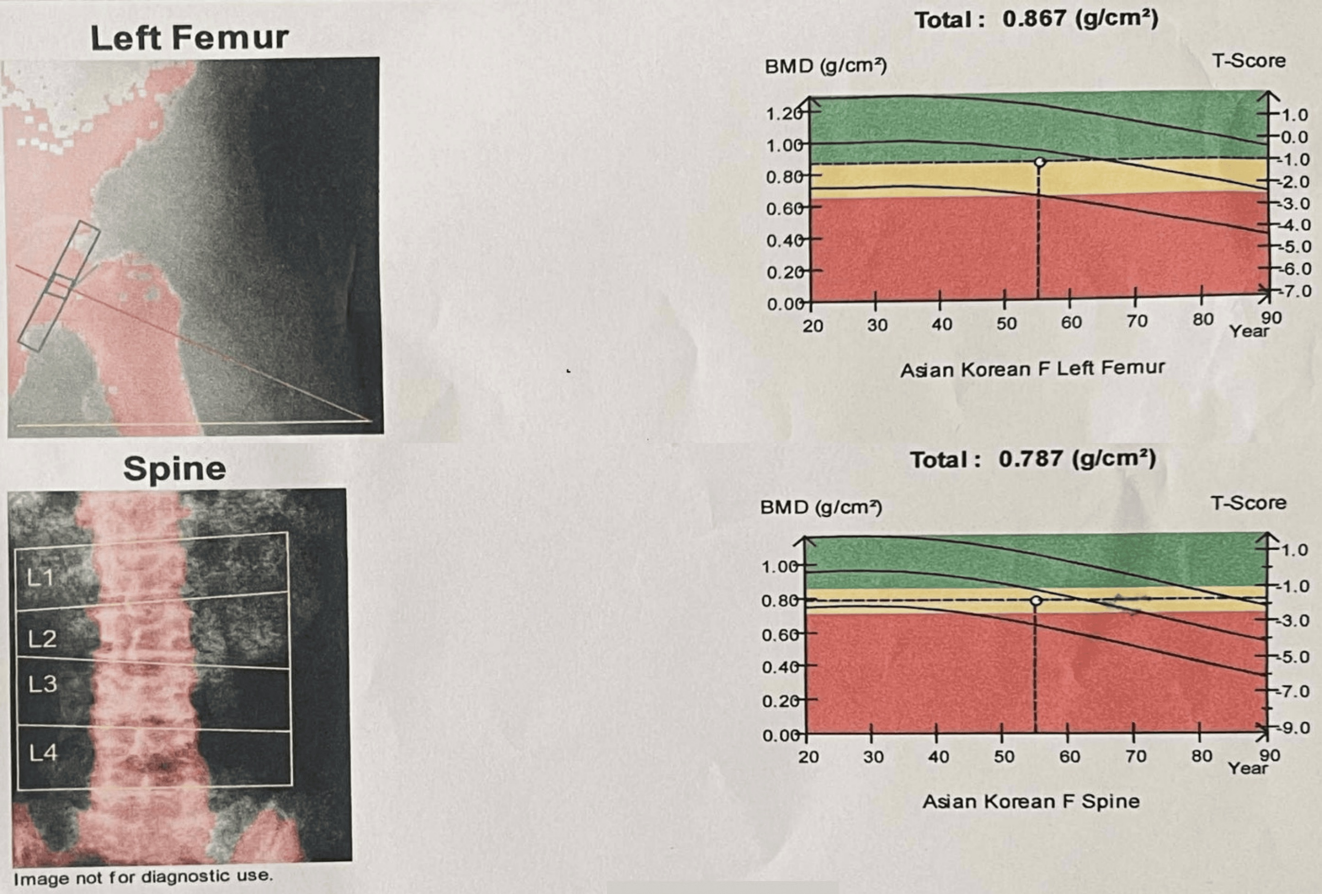
Bone mineral density result

Initial laboratory findings showed a serum potassium level as low as 1.66 mmol/L. Arterial blood gas analysis revealed normal anion gap metabolic acidosis (bicarbonate (HCO₃⁻) 15.4 mmol/L, pH 7.235, pCO₂ 37.3 mmHg), serum sodium 149.1 mmol/L, and serum chloride 118 mmol/L. Urinalysis showed alkaline urine (pH 8.0) with low specific gravity (1.006). Urinary electrolytes revealed sodium levels of 73.7 mmol/L, potassium of 11.96 mmol/L, and chloride of 76 mmol/L (Table 1). Based on the laboratory findings, the urinary potassium-to-creatinine ratio = 15.53 mmol/mmol, the serum anion gap = 15.7, and the urine anion gap = 9.66 can be readily calculated. These findings led to a diagnosis of distal RTA (type 1 RTA). Ultrasound examination of the patient’s parotid glands revealed no abnormalities (Figure 1). Bone mineral density assessment by dual-energy X-ray absorptiometry (DEXA) scan indicated osteopenia, with T-scores of -1.1 at the femoral neck and -1.8 at the lumbar spine (Figure 2 and Table 2).

**Table 1:** Laboratory results HCO_3_-: bicarbonate; HCV: hepatitis C virus; ELISA: enzyme-linked Immunosorbent assay

Lab	Value	Reference range
Serum		
Sodium (mmol/L)	149.1	135 – 145
Potassium (mmol/L)	1.66	3.5 – 5.0
Chloride (mmol/L)	118	96 – 110
HCO3- (mmol/L)	15.4	21 – 26
pH	7.235	7.35 – 7.45
pCO_2_ (mmHg)	37.3	35 – 45
Creatinin (µmol/L)	80.9	Male: 62 – 106; Female: 44 - 80
Urine		
pH	8.0	4.8 -7.4
Specific gravity	1.006	1.015 – 1.025
Sodium (mmol/L)	73.7	40 – 220
Potassium (mmol/L)	11.96	25 – 125
Chloride (mmol/L)	76	110 – 250
Creatinine µmol/L	770,9	
Protein (g/24h)	0.0744	< 0.15
Immunologic workup		
Anti–HCV	Negative	Negative
Anti-HIV	Negative	Negative
C3 (mg/dL)	127	90 – 180
C4 (mg/dL)	12.8	10 – 40
Direct Coombs test	Posivite	Negative
Indirect Coombs test	Positive	Negative
ANA (ELISA)	Negative	< 0.9: Negative; 0.9 – 1.1: gray zone; > 1.1: Positive
Anti–DSDNA	Negative	Negavite: 0 – 5; Borderline: 6 – 10; Positive: 11 – 25, 26 – 50; Strong positive: > 50
Anti–SSA	Positive (49)	Negavite: 0 – 5; Borderline: 6 – 10; Positive: 11 – 25, 26 – 50; Strong positive: > 50
Anti–Ro 52	Strong positive (83)	Negavite: 0 – 5; Borderline: 6 – 10; Positive: 11 – 25, 26 – 50; Strong positive: > 50
Anti–SSB	Strong positive (67)	Negavite: 0 – 5; Borderline: 6 – 10; Positive: 11 – 25, 26 – 50; Strong positive: > 50
Anti–RNP	Positive (20)	Negavite: 0 – 5; Borderline: 6 – 10; Positive: 11 – 25, 26 – 50; Strong positive: > 50
Anti–Sm	Negative	Negavite: 0 – 5; Borderline: 6 – 10; Positive: 11 – 25, 26 – 50; Strong positive: > 50
Anti–Scl–70	Negative	Negavite: 0 – 5; Borderline: 6 – 10; Positive: 11 – 25, 26 – 50; Strong positive: > 50
Anti-Jo-1	Negative	Negavite: 0 – 5; Borderline: 6 – 10; Positive: 11 – 25, 26 – 50; Strong positive: > 50
Rheumatoid factor (IU/mL)	32	< 8

**Table 2:** Densitomety data N/C: not calculated

Region of interest	Bone mineral density (g/cm^2^)	Bone mineral content(g)	Area (cm^2^)	T – Score	Z – Score
Left femur					
Neck	0.761	2.21	2.90	- 0.5 (94%)	0.6 (109%)
Greater trochanter	0.739	0.25	0.34	0.7 (110%)	1.4 (122%)
Intertrochanteric region	0.883	18.98	21.49	- 1.1 (84%)	- 0.7 (90%)
Total hip	0.867	21.44	24.74	- 1.1 (85%)	- 0.6 (92%)
Ward	0.876	0.65	0.74	N/C	N/C
Spine					
L1	0.622	7.53	12.10	- 2.4 (71%)	- 1.4 (81%)
L2	0.748	10.16	13.59	- 1.9 (79%)	- 0.8 (89%)
L3	0.832	13.20	15.87	- 1.6 (83%)	- 0.5 (94%)
L4	0.904	13.93	15.41	- 1.3 (87%)	- 0.2 (98%)
Total	0.787	44.82	56.98	- 1.8 (81%)	- 0.7 (92%)

​​​The presence of anti-Ro/SSA and anti-La/SSB antibodies, along with sicca symptoms and parotid gland enlargement, was highly suggestive of primary Sjögren’s syndrome. However, because objective diagnostic tests such as salivary flow measurement, Schirmer’s test, and salivary gland biopsy could not be performed, the patient was classified as presumed primary Sjögren’s syndrome rather than receiving a definitive diagnosis.

Although the patient exhibited symptoms of dry eyes and mouth, she did not feel the need for further intervention. She had no other systemic manifestations aside from renal tubular abnormalities. Therefore, the patient was treated with oral potassium chloride and sodium bicarbonate (1 mmol/kg/day), along with oral calcium carbonate supplementation. Ten days later, her clinical condition was stable and serum potassium had normalized (3.95 mmol/L).

## Discussion

Hypokalemia, defined as a serum potassium concentration < 3.5 mmol/L, has a variable prevalence, approximately 2.7% in the general population [3] and up to 12% among hospitalized patients [4]. The prevalence may be as high as 56% in patients with primary hyperaldosteronism [5]. Severe hypokalemia (< 2.5 mmol/L) can lead to symptoms such as palpitations, muscle weakness, and even paralysis. Even asymptomatic mild hypokalemia is associated with increased risk of cardiovascular events and all-cause mortality [6].

**Figure 3:**
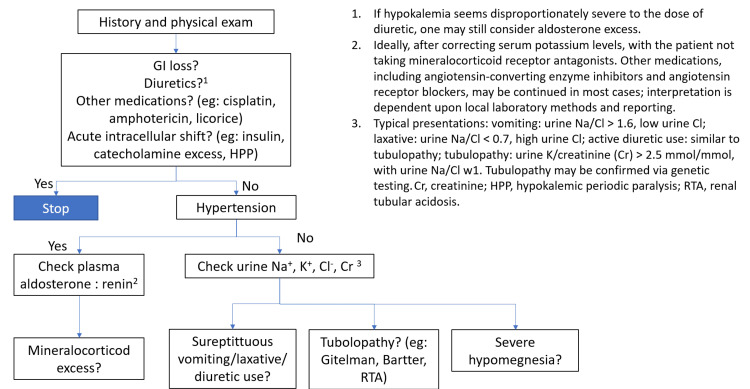
Pratical approach to hypokalemia HPP: hypokalemic periodic paralysis; Na+: sodium ion; K+: pottassium ion; Cl: chloride; RTA: renal tubular acidosis

Etiologies of hypokalemia are typically categorized into (1) decreased intake, (2) intracellular shift, and (3) increased loss (renal or extrarenal). Various diagnostic approaches have been proposed to evaluate hypokalemia. Among them, the Kidney Disease: Improving Global Outcomes (KDIGO) algorithm offers a practical, structured framework for identifying the underlying cause (Figure 3) [7]. Applying this approach allowed for the identification of distal RTA (type 1 RTA) as the cause of hypokalemia in our patient.

Metabolic acidosis can result from increased acid production, bicarbonate loss, or impaired acid excretion due to renal dysfunction. Chronic metabolic acidosis can lead to complications such as muscle wasting, decreased bone density, and progression of chronic kidney disease (CKD) [8]. Acidosis is classified into two main types based on the anion gap: (1) high anion gap and (2) normal anion gap metabolic acidosis. The latter may be due to extrarenal causes (most commonly chronic diarrhea) or renal causes (i.e., renal tubular acidosis) [9]. Differentiation is based on the renal response to acidosis. While direct measurement of urinary ammonium excretion is ideal, it is not widely available. Instead, urinary anion gap or urine osmolal gap can be used as surrogate markers [10]. In our patient, an alkaline urine pH of 8 and low serum potassium during metabolic acidosis were consistent with type 1 RTA, a defect in distal nephron proton excretion [9]. Classic features include hypokalemia, normal anion gap metabolic acidosis, and persistently high urine pH (>5.5). Associated findings include hypercalciuria and hypocitraturia, predisposing to nephrolithiasis and bone disease. Etiologies include genetic and acquired causes, with Sjögren’s syndrome being the most common acquired cause [9].

Upon confirming the diagnosis of type 1 RTA, additional evaluation suggested underlying primary Sjögren’s syndrome. As previously noted, primary Sjögren’s is a prototypical autoimmune disease with highly variable presentations. It predominantly affects women, with most cases diagnosed around the age of 50 [11]. The classic sicca syndrome involves dryness of the eyes and mouth. Diagnosis requires a combination of clinical evaluation (Schirmer’s test, salivary flow measurement), serologic testing (ANA, RF, anti-Ro, anti-La), and sometimes minor salivary gland biopsy [2]. The 2016 American College of Rheumatology/European League Against Rheumatism (ACR/EULAR) classification criteria for primary Sjögren’s syndrome [12] provide a standardized diagnostic framework. Although our patient did not meet full classification criteria, the presence of anti-Ro/SSA and anti-La/SSB antibodies, along with sicca symptoms and parotid gland enlargement, strongly supported the diagnosis. The disease generally follows a slow, benign course but can be associated with complications such as lymphoma or cardiovascular disease [1]. Renal involvement occurs in approximately 5% of cases [2], most commonly presenting as tubulointerstitial nephritis. Other possible renal manifestations include type 1 RTA with hypokalemia, Fanconi syndrome, and nephrogenic diabetes insipidus. Primary Sjögren’s syndrome affects the kidney through lymphocytic infiltration of renal tubules or immune complex deposition. Glomerular disease is less common [13]. A recent Taiwanese cohort study found that 5.8% of patients with Sjögren’s syndrome developed CKD, and 0.22% progressed to end-stage kidney disease (ESKD). Patients with Sjögren’s had a 49% increased risk of CKD compared to the general population, although the risk of ESKD was not significantly elevated (adjusted hazard ratio (HR) 0.82, 95% CI 0.58-1.16) [14].

In a patient such as this, differential diagnoses beyond Sjögren’s syndrome include HIV infection and sarcoidosis. Negative HIV serology effectively excludes HIV infection. In addition, the absence of respiratory symptoms makes sarcoidosis less likely. Furthermore, the presence of anti-SSA/SSB antibodies favors Sjögren’s syndrome rather than sarcoidosis [1]. Another point that warrants discussion in this patient is the positive Coombs test. Although a positive Coombs test is relatively uncommon in patients with primary Sjögren’s syndrome, this finding can be explained by disease pathogenesis, namely chronic B-cell activation and polyclonal hypergammaglobulinemia, leading to the production of anti-erythrocyte autoantibodies. In addition, a study by R. Ramakrishna has reported cases of Sjögren’s syndrome with a positive direct Coombs test [15]

Management is primarily symptomatic: artificial tears, increased fluid intake, and pilocarpine for sicca symptoms. According to the European Alliance of Associations for Rheumatology (EULAR) guidelines for the management of Sjögren’s syndrome, renal involvement should be treated based on whether it manifests as tubular or glomerular disease. In cases of tubular involvement, as in this patient, therapeutic decisions are guided by the EULAR Sjögren’s Syndrome Disease Activity Index (ESSDAI) category (low, moderate, or high). Patients with a low ESSDAI score require only supportive treatment with sodium bicarbonate and potassium supplementation, whereas glucocorticoids or other immunosuppressive agents, such as mycophenolate mofetil, azathioprine, or cyclophosphamide, may be indicated in those with moderate to high ESSDAI scores [16].

## Conclusions

This case illustrates the diverse clinical manifestations of primary Sjögren’s syndrome, with the initial presentation being lower limb weakness due to severe hypokalemia. A systematic approach to the evaluation of hypokalemia, including acid-base status, urine pH, urinary electrolytes, and anion gap analysis, led to the diagnosis of distal RTA. Careful history-taking, physical examination, and targeted immunologic testing guided the identification of Sjögren’s syndrome as the underlying etiology. This case underscores the importance of a structured diagnostic approach to electrolyte disturbances and the need to consider autoimmune causes in unexplained renal tubular dysfunction.
